# A Rare Case of Pericardial Effusion in a Patient with Silicosis

**DOI:** 10.1155/2019/5068580

**Published:** 2019-06-09

**Authors:** Kosuke Saku, Keisuke Yamamoto, Hironori Inoue, Masahiro Ueno

**Affiliations:** Department of Cardiovascular Surgery, Tenyokai Central Hospital and Central Clinic, Kagoshima, Japan

## Abstract

Silicosis is an occupational lung disorder caused by inhalation of silica dust. It not only causes respiratory disorders but also affects other organs. We report an extremely rare case of silicosis complicated by pericarditis in an 83-year-old male. He had been working as a coal miner and was diagnosed with silicosis at the age of 63. Because he had experienced repeated pericardial effusions, he was referred for a surgical pericardial biopsy to elucidate the cause of his repeated pericardial effusion and to perform pericardial fenestration. Thoracoscopic surgery was performed. The pericardium was resected, and a drain was placed in the left thoracic cavity. Histopathological examination revealed the pericardial degeneration due to silicosis, suggesting that pericarditis and pericardial effusion are related to silicosis. The operation was successful, and he experienced no recurrence of pericardial effusion at the 7-month follow-up.

## 1. Introduction

Silicosis is an irreversible and incurable lung disease caused by inhalation of dust containing crystalline silica particles [1] and is one of the most important occupational diseases in the world [1, 2]. Occupations such as mining, stone work, and sandblasting are associated with an increased risk of silicosis in individuals [2]. Silicosis is a form of pneumoconiosis and can be associated with lung cancer and disorders of other organs [3]. Here, we report an extremely rare case of silicosis complicated by pericarditis and pericardial effusion.

## 2. Case Presentation

The patient was an 83-year-old male who had been working as a coal miner and was diagnosed with silicosis at another hospital at the age of 63. He had experienced repeated pericardial effusions 5 years ago and had undergone pericardiocentesis twice; however, the cause of pericardial effusion remained unclear, and he was referred to our hospital for surgical pericardial biopsy and pericardial fenestration.

**Figure 1:**
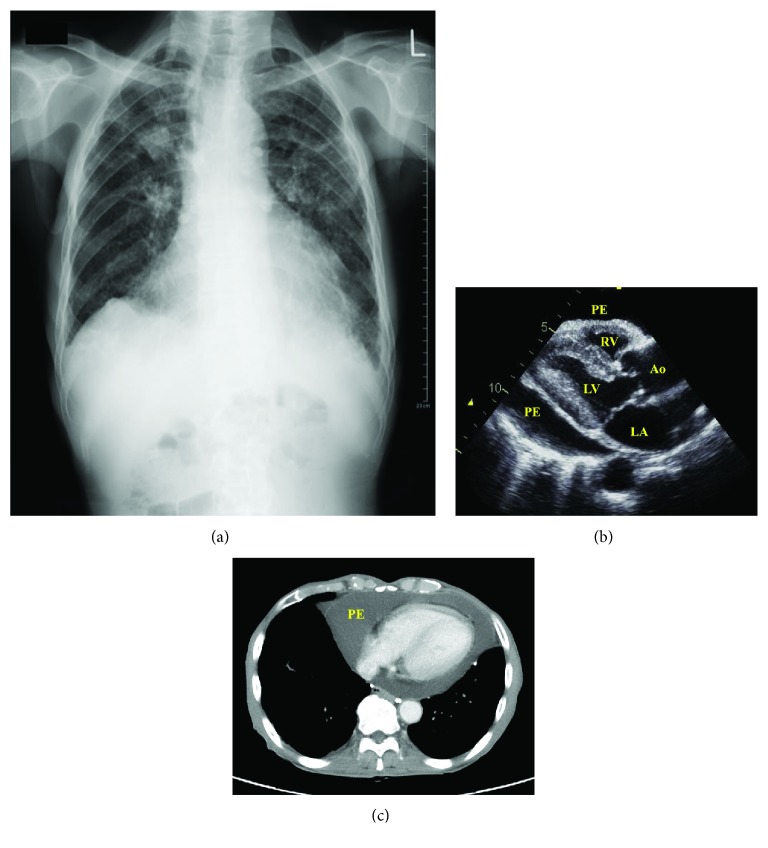
(a) Chest radiograph showing nodular opacities in the bilateral upper lung and diffuse small nodules throughout the lung field. (b) Transthoracic echocardiogram revealing a massive pericardial effusion. PE: pericardium effusion; LA: left atrium; LV: left ventricle; Ao: aorta; RV: right ventricle. (c) Computed tomography revealing a massive pericardial effusion. PE: pericardium effusion.

He presented with general fatigue and exhibited stable vital signs. Fine crackles were heard during inspiration, and a restrictive pattern was observed on pulmonary function testing (vital capacity 2040 mL (71% predicted) and forced expiratory volume in 1 s 1550 mL (81.2% predicted)). We noted peripheral vein swelling and mild limb edema. Laboratory testing showed a white blood cell count of 4900/*μ*L, hemoglobin levels of 11.3 g/dL, platelet count of 18.2 × 10^3^/*μ*L, and C-reactive protein levels of 0.06 mg/dL. No obvious liver or renal insufficiency (aminotransferase levels of 17 U/I, alanine transaminase levels of 6 U/I, total bilirubin levels of 0.2 mg/dL, total protein levels of 6.6 g/dL, albumin levels of 3.9 g/dL, blood urea nitrogen levels of 30.3 mg/dL, and creatinine levels of 0.8 mg/dL) was detected. His brain natriuretic peptide levels were 104.0 pg/mL. Low QRS voltage was observed on electrocardiography. A chest radiograph revealed cardiomegaly; his cardiothoracic ratio was 61.0% (Figure 1(a)). Opaque nodules measuring ~30 mm were observed bilaterally in the upper lung, and diffuse, small nodules were observed throughout the lung field and hilar region (Figure 1(a)). Transthoracic echocardiography and chest computed tomography both revealed massive pericardial effusion (Figures 1(b) and 1(c)). Additional findings on echocardiography included not only a right atrium collapse but also a hyperechoic pericardium and pericardial thickening (Figure 1(b)). In addition, there were no significant valvular disease and signs of heart failure (ejection fraction value of 84.0%, left ventricular end diastolic/systolic diameter of 39/18 mm, inferior vena cava diameter during inspiration of 18 mm, right ventricular systolic pressure of 29 mmHg, and *E*/*e*′ value of 15.1). The patient was referred for a surgical pericardial biopsy to elucidate the cause of his repeated pericardial effusion and to perform pericardial fenestration.

**Figure 2:**
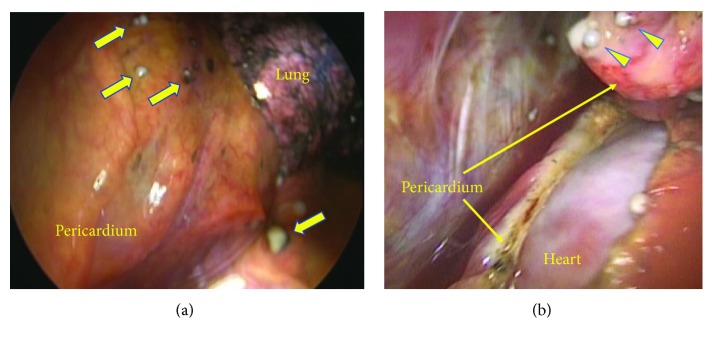
(a) Multiple white, nodular deposits (yellow arrows) observed throughout the pericardium. (b) Pericardial fenestration was performed using the Harmonic Scalpel®. White nodular-like deposits (triangle) were found throughout the pleura and pericardium.

Thoracoscopic surgery was performed using a 3 cm incision through the 8th intercostal space. Thoracoscopic examination again revealed serous pleural effusion and multiple nodular opacities throughout the pleura and pericardium (Figures 2(a) and 2(b)). A small incision was made on the pericardium (Figure 2(b)), taking care to preserve the left phrenic nerve, through which approximately 1200 mL of the effusion fluid was drained. We resected the pericardium largely of a 4 × 6 cm section using the Harmonic Scalpel® (Ethicon, US) and placed a drain in the left thoracic cavity.

**Figure 3:**
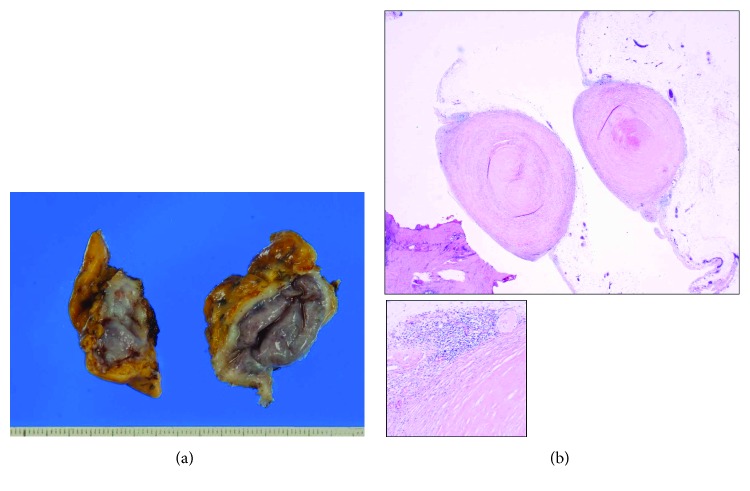
(a) Macroscopic findings of the resected pericardium. (b) Hematoxylin and Eosin staining revealed chronic infiltration of inflammatory cells and hyalinized fibrosis within the white nodular tissue of the resected pericardium.

Cytological examination of the pericardial biopsy sample was unremarkable; however, histopathological examination revealed inflammatory cell (mainly lymphocytes) infiltration and hyalinized fibrosis within the nodular tissue (Figures 3(a) and 3(b)), consistent with a diagnosis of silicosis. Lastly, polymerase chain reaction (PCR) results were negative for tuberculosis.

The patient's fatigue was resolved after surgery, and he experienced no recurrence of pericardial effusion at the 7-month follow-up.

## 3. Discussion

Silicosis is a well-known occupational respiratory disease [1], which causes lung inflammation and fibrosis. Silicosis is caused by inhalation and deposition of large amounts of crystalline silica over time [4]. Although protective measures such as dust control and respirators have reduced deaths attributable to silicosis in developed countries, new outbreaks still occur [5].

When silica dust is inhaled, the particles deposit within the distal airways. Macrophages ingest these particles and initiate an inflammatory response by releasing proinflammatory molecules such as tumor necrosis factor, interleukin-1, and other cytokines. These often lead to tissue fibrosis and formation of nodular lesions [5]. Thus, silicosis is characterized by fibrotic nodules with concentrically arranged collagen fibers, central hyalinization, and fibrotic lesions [5]. In our case, white nodules were showed on the pericardial surface, and inflammatory cell infiltration and hyalinized fibrosis within the nodular tissue were found by the histopathological examination. It was diagnosed as silicosis nodules.

Although the deposition of silica is common in lung tissues, it can also accumulate in other organs like the brain, peritoneum, bone marrow, liver, and spleen [6]. Our patient presented with an extremely rare condition as silica affected the pericardium on evoking a chronic inflammatory response; these processes could be involved in pericarditis and recurrent pericardial effusion.

There are a few reports on silica involved in pericardial degeneration [2, 6]. Jiang and Shao reported a male stone miner patient having both silicosis and constrictive pericarditis [2]. Mohebbi et al. reported a pericardial plague seen in a patient with silicosis and pathology findings of the pericardial specimen which had a typical basket-weave collagen pattern suggesting silica deposits [6]. The mechanisms through which silica causes pericarditis are unclear; however, one potential cause is immune reactions [2]. Individuals with silicosis manifest a significantly increased risk for autoimmune diseases such as rheumatoid arthritis, progressive systemic sclerosis, systemic lupus erythematosus, ANCA-associated small vessel vasculitis, and Wegener's granulomatosis [7]. It was considered that silica deposits broke the immune homeostasis [2, 7]. In addition, silica which has the nondegradable nature leads to saturation of the macrophages, and it has been demonstrated that macrophages that ingest silica release factors that increase biosynthesis by fibroblasts [7, 8]. Zeren et al. have reported that lymphatic and venous spread of the dust is thought to be responsible for extrapulmonary disease, because silica particles cannot be destroyed with phagocytosis and enzymatic reactions [3]. These immune responses may have affected not only the lungs but also the pericardium in our case. Another possibility is unrelated, yet concurrent, infections leading to pericarditis [2]. Exposure to silica dust is a potent risk factor for tuberculosis, as demonstrated by multiple studies [9]. However, the PCR result for tuberculosis was negative in our patient.

Because pericardial fenestration provides continuous drainage into the pulmonary cavity, absorption from the pleura is expected [10]. Consequently, pericardial fenestration carries a lower risk of pericardial effusion recurrence than temporary drainage methods like pericardiocentesis. A previous study has described that pericardial fenestration or pericardial biopsy via thoracoscopy is a safe and minimally invasive technique [10] and that the thoracoscopy approach can excise a larger window of the pericardium to prevent a recurrent effusion [10]. Our patient also achieved good outcomes following pericardial fenestration via thoracoscopy.

## 4. Limitations

This case report has some limitations. (1) Although histopathological examination has revealed that the degeneration of the pericardium is due to silicosis, the silica itself has not been identified from the pericardium. In addition, histopathological examination revealed chronic inflammatory cell (mainly lymphocytes) infiltration and hyalinized fibrosis within the nodular tissue, and it was diagnosed as a consistent finding as silicosis nodules. (2) The mechanisms through which silica causes pericarditis are still unclear. Further studies are needed in the future.

## 5. Conclusion

We reported an extremely rare case of silicosis complicated by pericarditis and pericardial effusion. The precise mechanisms through which silica deposits affect the pericardium are unclear; however, pericarditis and pericardial effusion could be the products of immune reactions induced by silica and/or translocation of silica dust via lung tissue. Although the pathological condition in which silicosis is involved in pericarditis as this case is very rare, we should be aware of such a pathological condition in the clinical practice.
